# Risk-reducing salpingo-oophorectomy: a meta-analysis on impact on ovarian cancer risk and all cause mortality in BRCA 1 and BRCA 2 mutation carriers

**DOI:** 10.1186/s12905-014-0150-5

**Published:** 2014-12-12

**Authors:** Claudia Marchetti, Francesca De Felice, Innocenza Palaia, Giorgia Perniola, Angela Musella, Daniela Musio, Ludovico Muzii, Vincenzo Tombolini, Pierluigi Benedetti Panici

**Affiliations:** Department of Gynecological and Obstetrical Sciences and Urological Sciences, University of Rome 2“Sapienza”, Viale del Policlinico, 155, 00161 Rome, Italy; Department of Radiotherapy, Policlinico Umberto I “Sapienza” University of Rome, Viale Regina Elena 326, 00161 Rome, Italy

**Keywords:** Risk-reducing salpingo-oophorectomy, Prophylactic, BRCA, Ovarian cancer, Cause, Meta-analysis

## Abstract

**Background:**

Women with BRCA1 and BRCA2 mutation carriers are at substantially elevated risk of developing ovarian cancer. The aim of the meta-analysis is to clarify the role of risk-reducing salpingo-oophorectomy (RRSO) to reduce ovarian cancer risk and mortality in women with BRCA 1 and BRCA 2 mutation carriers.

**Methods:**

Pubmed, Medline and Scopus were searched to select English-language articles. Two investigators independently extracted characteristics and results of selected studies. Articles were included only if prospective and if absolute numbers of ovarian cancer and death events were available or derivable from the test. Pooled hazard ratio (HR) with 95% confidence interval (CI) was calculated using fixed effects model.

**Results:**

Meta-analysis of 3 prospective studies demonstrated a significant risk reduction of ovarian cancer with RRSO in BRCA 1 and BRCA 2 mutation carriers, as well as benefit in all-causes mortality incidence.

**Conclusions:**

It may be justified to recommend RRSO to reduce ovarian cancer risk and all-causes mortality in women with a mutation in BRCA 1 and BRCA 2.

## Background

Women with a germline mutation in BRCA 1 and BRCA 2 genes are at substantially elevated lifetime risk of developing ovarian cancer (15% – 56%) than the general population (1.4%) [1–3]. Understanding genetic basic mechanisms of disease has allowed the development of primary prevention and risk-reducing salpingo-oophorectomy (RRSO) was introduced with the aim of reducing risk ovarian cancer (OC). Recently, empirical data confirms this hypothesis, demonstrating OC reduction risk of 85% to 95% in these patients [4]. Nowadays, women with BRCA 1 and BRCA 2 mutations are therefore strongly advised to have prophylactic surgery once childbearing is complete [4].

The aim of this meta-analysis is to report the outcomes of homogeneous prospective studies in order to define conclusive results of RRSO impact in ovarian cancer incidence and all-causes mortality and to help clinicians and women in making cancer risk reduction decisions.

## Methods

### Data extraction and studies selection

The Preferred Reporting Items for Systematic Reviews and Meta-Analyses (PRISMA) statement was followed to perform the meta-analysis. It includes studies without any restrictions on publication date. The last search was done on July 2014. Literature electronic databases (Pubmed, Medline and Scopus) were searched for “oophorectomy”, “salpingo-oophorectomy”, “prophylactic oophorectomy”, “risk-reducing salpingo-oophorectomy”, “ovarian cancer” and “BRCA” in title and abstract. Studies that compared preventive oophorectomy with follow-up policy in women with a mutation in BRCA 1 and BRCA 2 were eligible. Prospective studies, written in English, were included. Reference lists of previously published reviews and meta-analyses were explored. Review articles, case reports, commentaries and letters were not included. Conference abstracts were not considered because of the insufficient data provided by the authors.

Two independent reviewers (CM and FDF) selected the identified studies based on the title and abstract. If the study’s topic could not be ascertained from its title or abstract, the full-text version would be retrieved for evaluation. Disagreement was resolved by discussion or consensus or with a third researcher (LM).

Studies were eligible if patients had a proven mutation status, were cancer-free at study enter and had not previous history of prophylactic surgery.

In the closer evaluation of potentially eligible articles, because large collaborations are needed to study BRCA 1 and BRCA 2 carriers, many of the studies had overlapping centers. When two articles appeared to report results with overlapping data, only the data representing the most recent publication or with the larger sample size were included in the meta-analysis. Although we made every attempt to eliminate redundancy in data represented in our meta-analysis, we cannot rule out the possibility that a few individuals participated on more than one study. From all including studies were obtained: first author’ surname, publication year, sample size of cases and controls, treatment, duration of follow-up, detection rate.

For the subgroup analysis of OC risk reduction according with gene-specific mutation, we chose the first experience published by Finch *et al.* [5] in order to evaluate the mutation specific data.

### End-points

Primary end-point was the risk of developing OC; secondary end-point was the impact on all-causes mortality. Moreover, all-causes mortality incidence was studied in those patients who had or had not a history of breast cancer; whereas, in these women, data analysis of OC risk was excluded because there were dishomogeneous data.

A subgroup analysis – in patients BRCA1 and BRCA2 mutated – was performed; this data analysis concern two studies (4310 patients) but, as above mentioned, due to lack of specific data in the update Finch *et al.* study [6], we considered the previous one [5].

### Statistical analysis

Cancer risk and mortality analysis were stratified by studies and hazard ratio (HR). The pooled HR was calculated using a fixed- or a random- effect models. Forest plot were used for graphical representation of each study and pooled analysis.

The size of every box represents the weight that the corresponding study exerts in the meta-analysis; confidence intervals of each study are displayed as horizontal line through the box.

The pooled HR is symbolized by a solid diamond at the bottom of the forest plot and the width of the square represents the 95% CI of HR. HR, variance, 95% CI, log [RR] and SE for each study were extracted or calculated based on the published studies according to the methods described by Tierney in 2007 [7]. A significant two-way p-value for comparison was defined as p <0.05. Statistical heterogeneity between studies was examined using both the Cochrane Q statistic (significant at p <0.1) and the I^2^ value (significant heterogeneity if >50%) [8]. Statistical analysis was performed by Review Manager 5.0 (http://www.cochrane.org). Publication bias was examined using analyses described by Egger and Begg [9,10].

## Results

**Figure 1:**
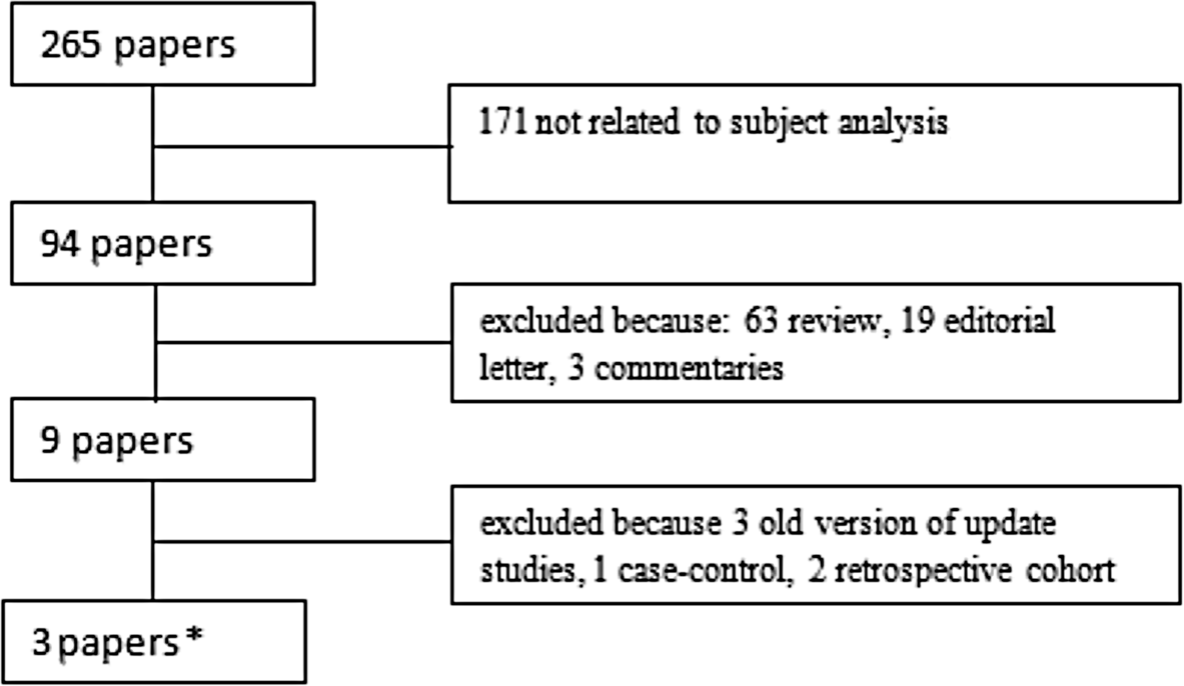
**Flow chart of meta-analysis.**

The literature search identified a total of 265 potentially relevant papers. Articles were excluded because of subject not related to the study (n = 171), review (n = 63), editorial letter (n = 19). Three articles were eliminated because have updated versions, whereas several studies were excluded because retrospective (2 articles) and case-control (1 article) [5,11,12]. Three prospective studies (9192 patients) were included in the final analysis (Table 1) [6,13,14]. Flow chart of meta-analysis is shown in Figure 1. Mean follow-up is 4.0 years. The χ2 tests for heterogeneity of each comparison showed no significant heterogeneity. No significant publication bias was found.

**Table 1:** **Characteristic of prospective studies**

**Study (country)**	**Patients with/without RRSO**			**Follow-up (years)**	**Age at RRSO (mean)**
	***All***	***BRCA1***	***BRCA2***		
Kauff *et al.* [13] (USA)	509/283	325/173	184/110	3.4	47.1
Domchek *et al.* [10] (USA)	939/1678	681/1006	258/672	6.2	43.2
Finch *et al.* [6] (USA-Europe)	3513/2270	2649/1824	864/446	5.6	47.75

RRSO: prophylactic salpingo-oophorectomy.

### Risk-reducing salpingo-oophorectomy and ovarian cancer risk

**Figure 2:**
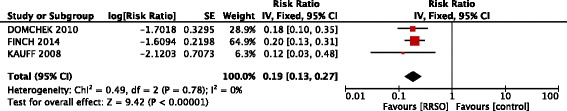
**Forest plots of relative risk (RR) estimates for risk reduction of ovarian cancer associated with risk-reducing salpingo-oophorectomy in the overall population of BRCA 1 and BRCA 2 mutation carriers.**

In all published studies, the RRSO consistently reduced OC risk over exclusive control. The OC risk after RRSO expressed as HR was 0.19 (95% CI: 0.13 – 0.27, p <0.00001) (Figure 2).

### Risk-reducing salpingo-oophorectomy and all-causes mortality

**Figure 3:**
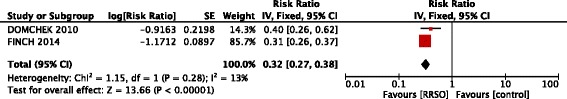
**Forest plots of relative risk (RR) estimates for all-causes mortality associated with risk-reducing salpingo-oophorectomy in the overall population of BRCA 1 and BRCA 2 mutation carriers.**

**Figure 4:**
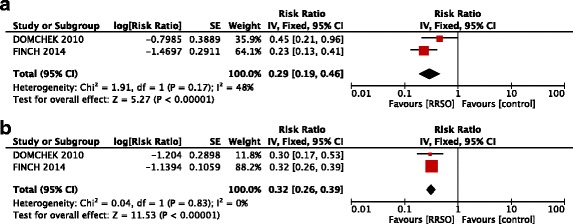
**Forest plots of relative risk (RR) estimates for all-causes mortality associated with risk-reducing salpingo-oophorectomy in BRCA 1 and BRCA 2 mutation carriers without prior (a) and with prior breast cancer (b).**

The all-cause mortality benefit associated with RRSO was 0.32 (95% CI: 0.27 – 0.38, p <0.00001) for all population (Figure 3). Among patients with or without previous breast cancer the risk reduction of RRSO was similar, with a modest benefit in patients without history of breast cancer: 0.29 (95% CI: 0.19 – 0.46, p <0.00001) versus 0.32 (95% CI: 0.26-0.39, p <0.00001) (Figure 4).

### BRCA subgroup analysis

The following analysis concerned two studies including 4310 patients with a mean follow-up of 4.8 years.

**Figure 5:**
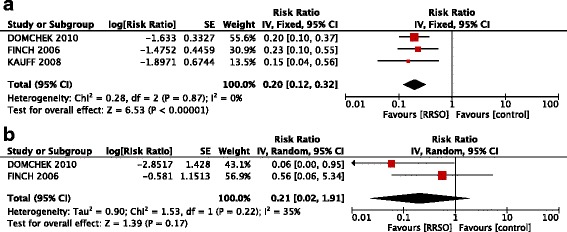
**Forest plots of relative risk (RR) estimates for risk reduction of ovarian cancer associated with risk-reducing salpingo-oophorectomy in BRCA 1 (a) and BRCA 2 (b) mutation carriers.**

The HR of OC risk reduction was significantly larger in BRCA 1 subgroup (0.20; 95% CI: 0.12 – 0.32, p <0.00001), whereas there was no significant benefit in BRCA 2 patients (0.21; 95% CI: 0.02 – 1.91, p =0.22 ) (Figure 5).

**Figure 6:**
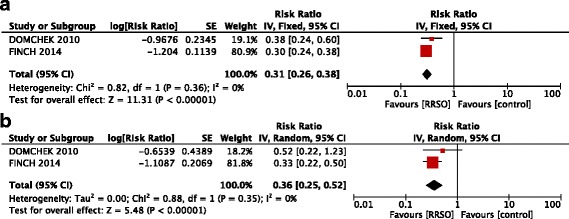
**Forest plots of relative risk (RR) estimates for all-causes mortality associated with risk-reducing salpingo-oophorectomy in BRCA 1 (a) and BRCA 2 (b) mutation carriers.**

All-cause mortality was equally strong for BRCA1 (HR, 0.31; 95% CI, 0.26 – 0.38, p <0.00001) and BRCA2 mutation carriers (HR, 0.36; 95% CI, 0.25 – 0.52, p <0.00001) (Figure 6).

## Discussion

Despite extensive research efforts, consisting of 3 prospective studies, 1 case control, 2 retrospective studies and 1 meta-analysis, the role of RRSO in reducing the risk of OC is still debated and safety concerns are still discussed [5,6,11,13–15].

Our study tries to address this problem using the standard methodology of meta-analysis, and formally assessing the presence and sources of heterogeneity in the results of the available studies. After the publication of the previous meta-analysis by Rebbeck *et al.* [15], two large-population studies have been published [6,13]. Therefore, a larger number of patients and exclusively prospective studies have been considered in our meta-analysis; consequently the statistical power has increased and we have been able to evaluate not only the OC risk reduction but also the all-cause mortality after RRSO: finally we evaluated the effect of RRSO in the overall population, as well as in BRCA 1 and BRCA 2 subgroups distinctly.

The importance of the observed reduction in the risk of OC, resulting from RRSO, did not modify compared to Rebbeck *et al*. [15] conclusions. Our results provide convincing evidence for support the efficacy of RRSO strategy with an HR of 0.19 (0.13 – 0.27), which means approximately 80% risk-reduction of OC. Importantly there was not heterogeneity between the studies (I =0%), indicating a strong accordance in the results of these prospective studies, and emphasizing the robustness of benefit observed.

This advantage was present in patients, having either BRCA 1 (HR 0.20; 95% CI, 0.12 – 0.32), or BRCA 2 (HR 0.21; 95% CI, 0.02 – 1.91) mutations even if in the latter group a small number of patients may affect the strength of evidences. The difference among BRCA 1 and BRCA 2 mutation carriers may also be explained by two main reasons. First of all, in literature is reported a low absolute number of BRCA 2-associated gynecologic cancers [14]. Secondly, it is now well established that among women with BRCA 2 mutation, the risk of gynecologic cancer is only 2% to 3% by the mean age of 50 years, while it increases in the late 30s in women with BRCA 1 mutation [2,16].

This meta-analysis also examines a new aspect of the RRSO: its impact on all-causes mortality. RRSO assured a clear benefit, reducing mortality, both in general population (HR, 0.32; 95% CI, 0.27 – 0.38) and in its BRCA subgroups (HR, 0.31; 95% CI, 0.26 – 0.38 and HR, 0.36; 95% CI, 0.25 – 0.52 for BRCA 1 and BRCA 2, respectively). The observation that RRSO has a profound protective effect on all-causes mortality allows significant reflections. Major concerns about RRSO procedure comes from several data referred to general population in which oophorectomy in women younger than 45 years is associated with increased mortality [17]. Nonetheless, even if we can assume that the risk/benefit ratio of RRSO is significantly different in BRCA 1 and BRCA 2 mutation carriers than in the general population, we should also admit that approximately 60% of women with a BRCA 1 or BRCA 2 mutation is elected to undergo RRSO between 35 and 40 years of age, thus before menopause [18,19]. RRSO may negatively influence patient’s quality of life and health, due to a dramatically rapid decline in estrogen and androgen levels [17,20]. Surgical menopause can result in severe hot flashes, vaginal dryness, sexual dysfunction, sleep disturbances and cognitive changes as well as increased risk of cardiovascular disease [21]. Therefore, even if our results could reassure clinicians on health benefits of RRSO (cancer prevention) also the risk of the procedure (quality of life and long-term sequelae) should be mentioned.

With this regard, data on safety and feasibility of hormonal replacement treatment (HRT) in oophorectomized patients with BRCA 1 and BRCA 2 mutation carriers are required. Despite the limitations of retrospective and prospective observational studies, short-term HRT seems to improve quality of life and, moreover, does not seem to have an adverse effect on oncologic outcomes in BRCA 1 and BRCA 2 mutation carriers without a personal history of breast cancer [22]. Prospective randomized studies concerning type, timing, and length of administration of HRT as well as its long-term effects on the association between RRSO and cancer risk in BRCA 1 and BRCA 2 mutation carriers are mandatory.

Our study may be accompanied by some limitations. Firstly, it was not possible to delineate a correct standardization by age of RRSO procedure. The mean age for RRSO was 46 years but a different age’s categorized analysis was not possible, because only Finch *et al.* [6] stratified results for age and the necessary data are missing. Secondly, even if we found larger samples size distinguishing BRCA 1 from BRCA 2 mutation carriers, information that can be drawn are still insufficient, not allowing definitive conclusions especially in BRCA 2 population. Thirdly, the mean follow up of the analyzed studies is of nearby 4 years; reasonably, longer follow up would be useful to better understand the impact of this procedure also in terms of quality of life. Finally, only studies of prospective nature have been analyzed because no randomized studies have been published in this setting. Even if it is clear that a randomized controlled study design would allow a better evaluation of risk reducing surgery on cancer risk and mortality reduction, it is generally accepted that a randomized approach would neither be acceptable nor ethical for the management of these patients and therefore, this field of research is limited to undertaking observational studies, with intrinsic methodological limitations [13].

Briefly, RRSO is highly effective in reducing OC, both BRCA 1 and BRCA 2 mutation carriers. Long-term follow up data as well as data from studies concerning the management of oophorectomized BRCA 1 and BRCA 2 mutation carriers patients are needed to further confirm.

## Conclusion

This meta-analysis provides an analysis of the benefit of RRSO – in term of ovarian cancer risk incidence and all-causes mortality – in patients with BRCA 1 and BRCA 2 mutation. Results could be used as reference data for clinical studies and clinical management.

## Abbreviations

RRSO: Risk-reducing salpingo-oophorectomy
HR: Hazard ratio
IC: Confidence interval
OC: Ovarian cancer
RR: Risk ratio
SE: Standard error
